# RETRACTION: Perineal Wound Complications After Vertical Rectus Abdominis Myocutaneous Flap and Mesh Closure Following Abdominoperineal Surgery and Pelvic Exenteration of Anal and Rectal Cancers: A Meta‐Analysis

**DOI:** 10.1111/iwj.70182

**Published:** 2025-01-12

## Abstract

**RETRACTION**: LiuJ. , FuC. , ChenZ. , and LiG. , “Perineal Wound Complications After Vertical Rectus Abdominis Myocutaneous Flap and Mesh Closure Following Abdominoperineal Surgery and Pelvic Exenteration of Anal and Rectal Cancers: A Meta‐Analysis,” International Wound Journal 20, no. 10 (2023): 3963–3973, 10.1111/iwj.14284.37539486 PMC10681467

The above article, published online on 04 August 2023, in Wiley Online Library (http://onlinelibrary.wiley.com/), has been retracted by agreement between the journal Editor in Chief, Professor Keith Harding; and John Wiley & Sons Ltd. Following an investigation by the publisher, all parties have concluded that this article was accepted solely on the basis of a compromised peer review process. In addition, the investigation found unattributed textual overlap between this article and another article by different authors (Buscail, et al. 2021 [https://doi.org/10.3390/cancers13040721]). The editors have therefore decided to retract the article. The authors disagree with the retraction.

**RETRACTION**: J. Liu , C. Fu , Z. Chen , and G. Li , “Perineal Wound Complications After Vertical Rectus Abdominis Myocutaneous Flap and Mesh Closure Following Abdominoperineal Surgery and Pelvic Exenteration of Anal and Rectal Cancers: A Meta‐Analysis,” International Wound Journal 20, no. 10 (2023): 3963–3973, 10.1111/iwj.14284.37539486 PMC10681467

The above article, published online on 04 August 2023, in Wiley Online Library (http://onlinelibrary.wiley.com/), has been retracted by agreement between the journal Editor in Chief, Professor Keith Harding; and John Wiley & Sons Ltd. Following an investigation by the publisher, all parties have concluded that this article was accepted solely on the basis of a compromised peer review process. In addition, the investigation found unattributed textual overlap between this article and another article by different authors (Buscail, et al. 2021 [https://doi.org/10.3390/cancers13040721]). The editors have therefore decided to retract the article. The authors disagree with the retraction.

